# Intracerebral Transplantation of Mesenchymal Stromal Cell Compounded with Recombinant Peptide Scaffold against Chronic Intracerebral Hemorrhage Model

**DOI:** 10.1155/2022/8521922

**Published:** 2022-07-31

**Authors:** Soichiro Takamiya, Masahito Kawabori, Tsukasa Kitahashi, Kentaro Nakamura, Yuki Mizuno, Hironobu Yasui, Yuji Kuge, Aki Tanimori, Yasuyuki Takamatsu, Kohei Yuyama, Hideo Shichinohe, Miki Fujimura

**Affiliations:** ^1^Department of Neurosurgery, Graduate School of Medicine, Hokkaido University, Sapporo, Japan; ^2^Bioscience & Engineering Laboratory, FUJIFILM Corporation, Kanagawa, Japan; ^3^Central Institute of Isotope Science, Hokkaido University, Sapporo, Japan; ^4^Department of Applied Veterinary Sciences, Graduate School of Veterinary Medicine, Hokkaido University, Sapporo, Japan; ^5^Department of Rehabilitation Science, Faculty of Health Sciences, Hokkaido University, Sapporo, Japan; ^6^Lipid Biofunction Section, Faculty of Advanced Life Science, Hokkaido University, Sapporo, Japan; ^7^Institute of Health Science Innovation for Medical Care, Hokkaido University Hospital, Sapporo, Japan

## Abstract

**Background:**

Due to the lack of effective therapies, stem cell transplantation is an anticipated treatment for chronic intracerebral hemorrhage (ICH), and higher cell survival and engraftment are considered to be the key for recovery. Mesenchymal stromal cells (MSCs) compounded with recombinant human collagen type I scaffolds (CellSaics) have a higher potential for cell survival and engraftment compared with solo-MSCs, and we investigated the validity of intracerebral transplantation of CellSaic in a chronic ICH model.

**Methods:**

Rat CellSaics (rCellSaics) were produced by rat bone marrow-derived MSC (rBMSCs). The secretion potential of neurotrophic factors and the cell proliferation rate were compared under oxygen-glucose deprivation (OGD) conditions. rCellSaics, rBMSCs, or saline were transplanted into the hollow cavity of a rat chronic ICH model. Functional and histological analyses were evaluated, and single-photon emission computed tomography for benzodiazepine receptors was performed to monitor sequential changes in neuronal integrity. Furthermore, human CellSaics (hCellSaics) were transplanted into a chronic ICH model in immunodeficient rats. Antibodies neutralizing brain-derived neurotrophic factor (BDNF) were used to elucidate its mode of action.

**Results:**

rCellSaics demonstrated a higher secretion potential of trophic factors and showed better cell proliferation in the OGD condition. Animals receiving rCellSaics displayed better neurological recovery, higher intracerebral BDNF, and better cell engraftment; they also showed a tendency for less brain atrophy and higher benzodiazepine receptor preservation. hCellSaics also promoted significant functional recovery, which was reversed by BDNF neutralization.

**Conclusion:**

Intracerebral transplantation of CellSaics enabled neurological recovery in a chronic ICH model and may be a good option for clinical application.

## 1. Introduction

Intracerebral hemorrhage (ICH) accounts for 6.5%–19.6% of all strokes with an annual incidence of 15.9–36.4 per 100,000 people [1, 2]. Although tissue plasminogen activator and mechanical thrombectomy have paved the way for treating ischemic stroke in the acute phase, currently, no approved therapy for ICH exists, leading to higher mortality and morbidity [3]. Although rehabilitation is often performed for chronic patients, the efficacy is reported to be mostly limited up to 6 months from the onset [4]; therefore, rehabilitation focuses on maintaining the status quo. Thereby, a novel therapeutic method that can facilitate neurological recovery is warranted.

In recent years, stem cells have attracted much attention due to their anti-inflammatory, neurogenesis, and neovascularization capacities, and are considered a promising therapy for restoring neurological deficits. Due to its high cell delivering capacity, intracerebral transplantation is preferred over intravenous or arterial transplantation in the chronic phase of stroke [5, 6]. However, the most appropriate location for cell administration has not yet been determined. In the chronic phase of ICH, hematoma is absorbed, and a cavity filled with cerebral fluid develops. Although previous studies reported both “peri” and “intra” hollow cavity transplantation [7–9], administering stem cells into the hollow cavity seems more reasonable than to the peri hollow cavity, since the targets for stem cell therapy exist around this cavity. However, the chance of cell survival needs to be considered because only approximately 0.1% of cells are reported to survive when transplanted into the hollow cavity of its low blood supply [7]. Administering a product of higher cell viability into the cavity may be an optimal option to achieve maximum efficacy.

CellSaic is a newly developed mosaic-like cell aggregate consisting of mesenchymal stromal cells (MSC) and recombinant peptide (RCP) *μ*-pieces. RCP is made from the RGD (Arg-Gly-Asp) motifs of human collagen type I, which allows high cell attachment ability and better penetration of oxygen and blood vessels, and hence results in excellent cell survival and engraftment. CellSaic has shown better cell survival and functional recovery than MSC alone in other disease models [10, 11]. We therefore investigated whether the stereotactic transplantation of CellSaic developed from rat or human MSC into the hollow cavity of a chronic ICH model could achieve better functional recovery and aimed to elucidate its mode of action.

## 2. Materials and Methods

A full description of the methods including the experimental design can be found in the online-only Data Supplement (available here) . Animal protocols were approved by the Animal Studies Ethics Committee of the Hokkaido University Graduate School of Medicine (approval number: 17-0065) and by the Animal Care Committee of FUJIFILM Corporation (approval number: A-1-190289, A-1-190316, and A-1-190396). All experimental procedures were conducted in accordance with the Institutional Guidelines for Animal Experimentation and the Guidelines for Proper Conduct of Animal Experiments by the Science Council of Japan.

### 2.1. Preparation of rBMSCs and rCellSaic

Rat bone marrow-derived MSCs (rBMSCs) were obtained from 10-week male transgenic Sprague–Dawley (SD) rats expressing green fluorescence protein (GFP) [12]. Ten rats were used to obtain GFP-positive MSC, and the cells were used for subsequent experiments between three and ten passages. The characteristics of the cells were similar to the ones previously described [13]. RCP *μ*-piece was provided by Fujifilm Corporation (Kanagawa, Japan). rCellSaic were prepared by mixing 2.5 × 10^6^ rBMSCs and 1 mg of RCP *μ*-piece and were co-cultured on a specially designed cell culture dish (Fujifilm, Kanagawa, Japan) consisting of 1,000 arrayed micropores for 69 h [10, 14]. 1,000 rCellSaic per dish were formed, and a single rCellSaic is theoretically equivalent to 2.5 × 10^3^ rBMSCs [10, 14].

### 2.2. In Vitro Assessment of rCellSaic

rCellSaics and rBMSCs were incubated in oxygen-glucose deprivation (OGD) conditions (1% O_2_ in glucose-free medium) for 52 h to evaluate the trophic factor secretion ability in hypoxia and poor nutrition. Brain-derived neurotrophic factor (BDNF), hepatocyte growth factor (HGF), vascular endothelial growth factor (VEGF), and glial cell line-derived neurotrophic factor (GDNF) were evaluated from supernatant by enzyme-linked immunosorbent assay (ELISA) [15], and exosomes were collected by super-centrifugation of supernatant followed by particle density analysis [16]. The rCellSaic or rBMSCs were recultured in the normal condition for assessing cell survival and proliferation [17], and time-lapse imaging was performed for CellSaics to chronologically observe cell proliferation.

### 2.3. Chronic ICH Model and rCellSaic Transplantation

The ICH model was produced by internal capsule injection of 0.2 U type IV collagenase for 8-week-old male SD rats [18]. Complete hematoma absorption and cavity formation were defined as chronic ICH, and rats were serially euthanized for up to 2 weeks, and hematomas were absorbed, and hematoma cavities were formed until 14 d after ICH by using collagenase (Supplementary Figure IA). On the following day, animals that showed left hemiparesis and circled toward the right side, caused by left hemi-spatial neglect, were included in the study; eight rats that could walk straight were excluded from further experiments. The absorption rate of hemorrhage was confirmed by examining brain slices 3, 7, and 14 d after ICH. Two weeks after ICH onset, the animals were randomly assigned to three groups (rCellSaic group, rBMSC group, or saline group) and underwent stereotactic injection of rCellSaics (equivalent to 1.0 × 10^6^ of rBMSCs), 1.0 × 10^6^ of rBMSCs, or saline, respectively, to the hollow cavity via the same coordinates where the collagenase was injected.

### 2.4. Functional and Histological Assessment

Neurological function was assessed using the modified neurological severity score (mNSS) up to 42 d after ICH [19], and animals were sacrificed for histological analysis [17]. Hematoxylin and eosin (H&E) staining of brain coronal sections 4 mm posterior to the bregma was used to assess brain atrophy, and immunohistochemistry staining for anti-GFP antibody was used to evaluate cell engraftments.

Brain hemispheric sections located 2–4 mm posterior to the bregma were collected 3 d after transplantation for quantifying the BDNF secretion potential of CellSaic by ELISA.

I-123-iomazenil (123I-IMZ) single-photon emission computed tomography (SPECT) was performed 1 and 4 weeks after transplantation to evaluate the integrity of neural receptors [20]. Regions of interest (ROIs) of 1 mm thickness were placed on the ipsilateral and contralateral hemispheres in the coronal sections 4 mm posterior to the bregma. The variation rates of the ipsilateral/contralateral ratio from the first scan (1 week) to the second scan (4 weeks) were calculated.

### 2.5. Preparation of Human MSC and hCellSaic

Commercially available human BMSCs (Lonza, Basel, Switzerland) were used. One mg of RCP *μ*-piece and 1.2 × 10^6^ hBMSCs were used to prepare hCellSaic with a same method as that of rCellSaic. Single hBMSC-CellSaic (hCellSaic) was equivalent to 1.2 × 10^3^ hBMSC. Due to the high mortality rate associated with the collagenase model in immunodeficient rats, the chronic ICH model was produced by injection into the internal capsule of 3.0 *μ*L of 7.5 mM ouabain octahydrate solution in 5–6-week-old male immunodeficient rats (SD F344/NJcl-rnu/rnu) [18, 21]. The animals were randomly assigned to two groups, hCellSaic or saline group, and were injected with hCellSaics (4.8 × 10^5^ of hBMSC) or saline, respectively, into the hematoma hollow cavity one week after ICH. Since F344/NJcl-rnu/rnu rats comprise the immunodeficiency model, the animals did not receive immunosuppressant drugs. Neurological assessments (mNSS) were performed up to 6 weeks after transplantation as previously described.

### 2.6. Neutralizing the Trophic Factor Secretion of CellSaic and Its Mode of Action

Human antibodies for BDNF, HGF, GDNF, and IgG isotype control were co-cultured with CellSaics for 24 h to suppress the secretion potential of trophic factors [22]. After removing antibody, the concentration of trophic factors was evaluated up to 5 days. Then, hCellSaic (4.8 × 10^5^ of hBMSC) or anti-BDNF-hCellSaic (4.8 × 10^5^ of hBMSC) was transplanted to the animal to evaluate the functional mechanisms of hCellSaic.

### 2.7. Statistics

All assessments were performed in a blinded fashion. The data are presented as the mean ± standard error of the mean. Statistical analyses were performed using JMP Pro 14 software (SAS Institute Inc., Cary, NC, USA). Two groups were compared using the Wilcoxon rank sum test. Two-way analysis of variance was also performed for comparison between hCellSaic and anti-BDNF-hCellSaic. The Kruskal–Wallis test followed by the Steel–Dwass test was performed for neurological evaluation in which three groups were involved. Statistical significance was set at *p* < 0.05.

## 3. Results

### 3.1. CellSaics Possess Higher Secretion Potential of Trophic Factors and Better Cell Survival in an OGD Environment

**Figure 1:**
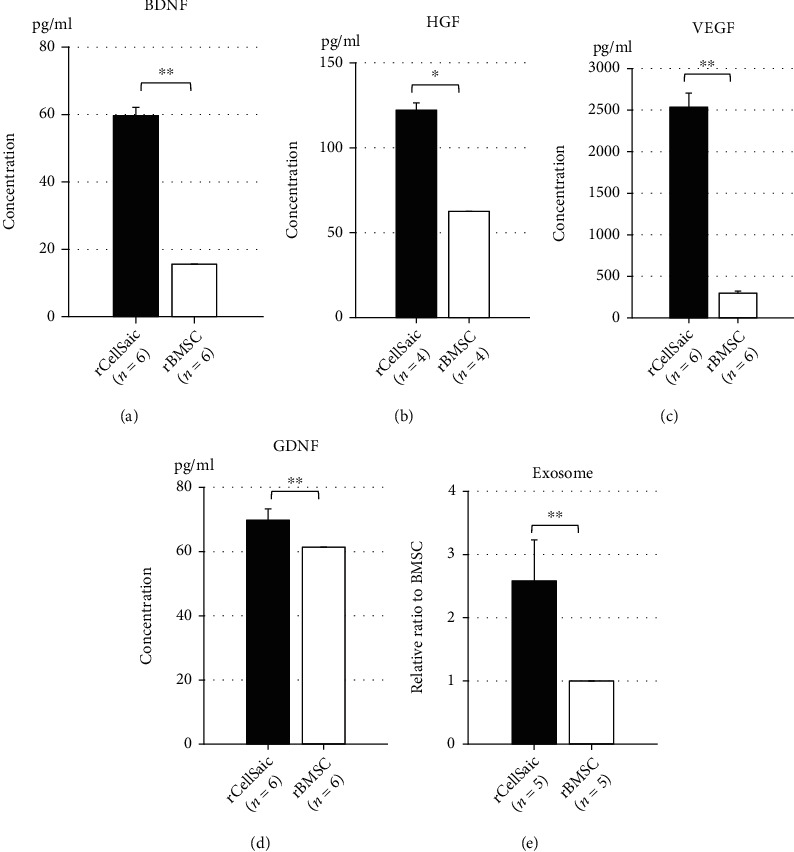
rCellSaics secreted more trophic factors and exosomes in the OGD condition and showed better cell proliferation. Neurotrophic factors and exosome secretion capacity under the OGD condition were compared between rCellSaic and solo-rBMSC. The concentrations of BDNF (a), HGF (b), VEGF (c), and GDNF (d) in CellSaic supernatants were significantly higher than those in solo-rBMSC. The exosomes released in the supernatants were significantly higher in rCellSaic than solo-rBMSC (e). Data are presented as the mean ± standard error (SE). ∗*p* < 0.05; ∗∗*p* < 0.01 (the Wilcoxon rank sum test).

**Figure 2:**
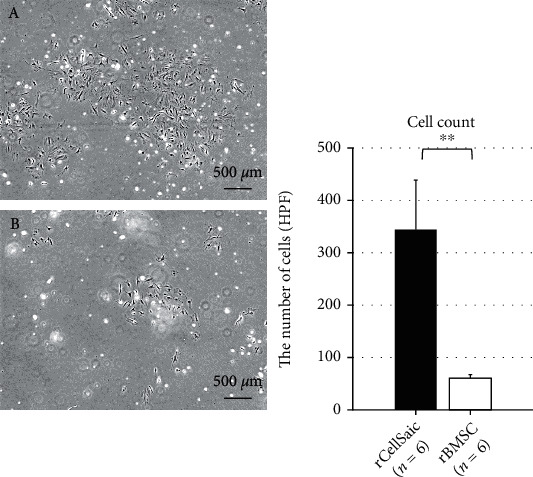
rCellSaics showed better cell proliferation in the OGD condition. CellSaic and solo-BMSC were recultured after incubation under an OGD condition. The average number of living cells 7 d after reculture was significantly higher in CellSaic (a) than in solo-rBMSC (b). Data are presented as the mean ± standard error (SE). ∗∗*p* < 0.01 (the Wilcoxon rank sum test).

Supernatants of rCellSaic and rBMSC were collected to evaluate the amounts of neurotropic factors released during OGD. The concentrations of BDNF, HGF, VEGF, and GDNF in rCellSaic supernatants were significantly higher than those in rBMSC alone (*p* = 0.0028, 0.0211, 0.0051, and 0.0096, respectively; Figures 1(a)–1(d)). rCellSaic also exhibited higher capacity for secretion of exosomes, with the number of exosome particles in rCellSaic being approximately two and a half times higher than that in rBMSC (*p* = 0.0075; Figure 1(e)). Furthermore, rCellSaics demonstrated better viability in an OGD environment than rBMSC. After incubation in an OGD environment, rCellSaics and rBMSC were cultured under normal conditions (Figure 2, Supplementary video). rBMSC contained in CellSaics migrated to the surrounding environment and proliferated (Figure 2(a)). In contrast, most rBMSC incubated solely in the OGD environment died and seldom adhered to the culture dish (Figure 2(b)). The average number of adhered and proliferated cells 7 d after reculture was significantly higher in the rCellSaic group than in the rBMSC alone group (*p* = 0.0132).

### 3.2. rCellSaic Transplantation into the Chronic ICH Cavity Improves Neurological Function

**Figure 3:**
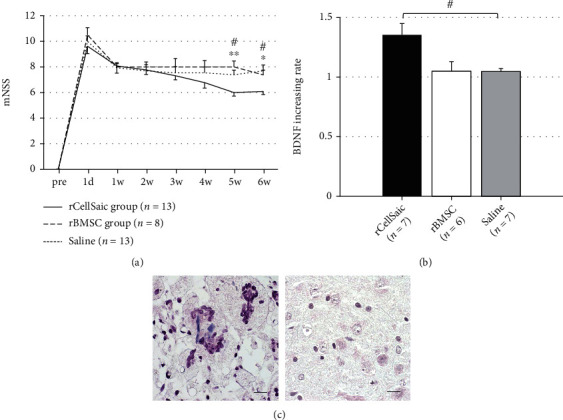
rCellSaic was associated with better neurological recovery, release of more trophic factors, and better cell engraftment after transplantation. The animals that received rCellSaic showed significantly better neurological recovery at 3 and 4 weeks after transplantation than the animals that received solo-rBMSC or saline (a). The cerebral local BDNF levels around ICH were compared three days after transplantation, and the rCellSaic group showed a significantly higher amount of BDNF compared with the saline group, and showed a higher trend than the rBMSC group (b). rBMSC derived from CellSaics engrafted in the host brain around the hollow cavity 4 weeks after the transplantation (c; left), whereas the cells could not be detected in the rBMSC groups (c; right) (magnification, ×400; scale bar = 50 *μ*m). Data are presented as the mean ± SE. #*p* < 0.05 vs. saline group; ∗*p* < 0.05 vs. rBMSC group; ∗∗*p* < 0.01 vs. rBMSC group (the Kruskal–Wallis test followed by the Steel–Dwass test).

A total of 74 rats were included in this study. Complete hematoma absorption and cavity formation were defined as chronic ICH. Rats were serially euthanized for up to 2 weeks, and hematomas were absorbed, and hematoma cavities were formed until 14 d after ICH (Supplementary Figure IA). The average diameter of the hollow cavities was 3.5 ± 0.6 mm × 2.5 ± 0.5 mm × 3.0 ± 0.5 mm, which was large enough to receive the transplantation through same coordinates. Thus, rCellSaics, solo-rBMSC, or saline were transplanted into the hollow cavity 2 weeks after the hemorrhage. The rCellSaic group showed significantly better neurological recovery than the rBMSC (*p* = 0.0097 and 0.0392, respectively) and saline (*p* = 0.0255 and 0.0277, respectively) groups at 3 and 4 weeks after transplantation (Figure 3(a)). No significant difference was found between the rBMSC and saline groups. Then, the amount of BDNF in the cerebral tissues around the hematoma cavity was measured to elucidate the mode of action of rCellSaic. Brain tissue transplanted with rCellSaic, rBMSC, or saline was harvested 3 days after transplantation. The rCellSaic group showed significantly higher BDNF than those in the rBMSC group and the saline group (*p* = 0.1306 and 0.0199, respectively; Figure 3(b)). These data indicate that rCellSaic can secrete a higher amount of BDNF after being transplanted to the hollow cavity. Immunohistochemistry using anti-GFP antibody revealed that BMSC derived from rCellSaics could be detected around the ICH cavity 4 weeks after transplantation in the rCellSaic group (Figure 3(c); left). In contrast, BMSC could not be detected in the rBMSC group (Figure 3(c); right).

### 3.3. Transplantation of rCellSaic May Prevent Brain Atrophy and Ameliorated the Damage of Neuronal Integrity

**Figure 4:**
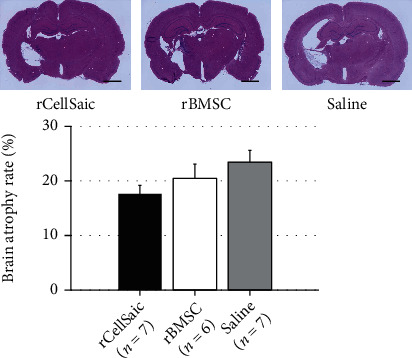
rCellSaics transplantation prevented brain atrophy. Brain sections of the rCellSaic, rBMSC, and saline groups were compared to evaluate brain atrophy (magnification, ×20; scale bar = 2 mm). The rCellSaic group showed a lower trend in the brain atrophy rate than the rBMSC and saline groups. (The Kruskal–Wallis test followed by the Steel–Dwass test).

**Figure 5:**
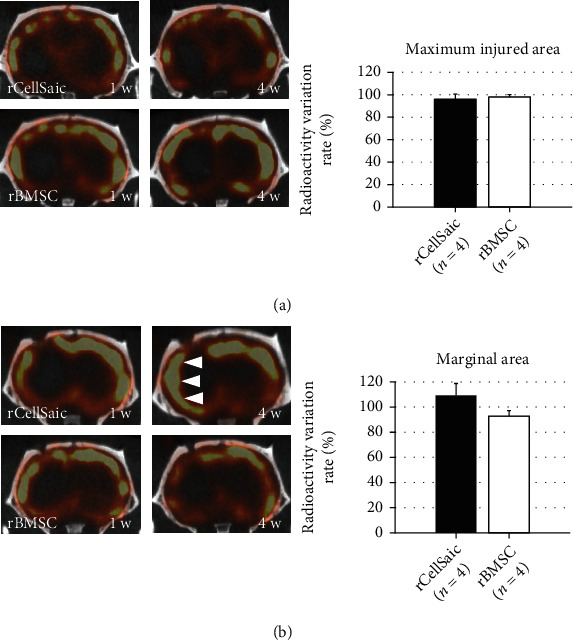
rCellSaics transplantation ameliorated neuronal integrity damage. Neuronal integrity was assessed by ^I-123^-iomazenil single-photon emission computed tomography. The radioactivity ratios were calculated, and their variation from 1 week to 4 weeks after transplantation in the rBMSC and rCellSaic groups was compared with respect to the maximum injured area of ICH (4 mm posterior to the bregma) (a) and the marginal area of ICH (2 mm posterior to the bregma) (b). While there was no difference in the radioactivity ratio variation between the two groups at the maximum injured area, the radioactivity ratio variation in the rCellSaic group showed a higher trend than that in the rBMSC group at the marginal area (b; arrow). The results indicate that benzodiazepine receptors were preserved or recovered at around the hematoma margin, which represents the neuro-integrity preservation potential of CellSaic. Data are presented as the mean ± SE (the Wilcoxon rank sum test).

Brain atrophy rates were further assessed between the groups. While all groups exhibited atrophy of the ipsilateral hemispheres and enlargement of lateral ventricles to some extent, the rCellSaic group had a lower trend of brain atrophy rate compared to the other groups (*p* = 0.0893; Figure 4), which indicates that rCellSaic was able to minimize brain damage. The change in neuronal integrity after rCellSaic and solo-rBMSC transplantation was further evaluated using ^123^I-IMZ SPECT, which detects the central benzodiazepine receptors expressed in neurons. Radioactivity was evaluated between 1 and 4 weeks after ICH to elucidate neural loss. Although there was no difference in the ratio of IMZ uptake around the maximum injured lesions in the rCellSaic and rBMSC groups (Figure 5(a)), the ratio of IMZ uptake around the marginal areas of ICH was increased in the rCellSaic group and decreased in the rBMSC group (*p* = 0.1124; Figure 5(b)). These results indicate that rCellSaic transplantation can better prevent neuronal loss and preserve neuronal integrity compared with transplanting solo-BMSC around the peri-damaged area.

### 3.4. hCellSaic Transplantation Can also Improve Neurological Function, which Is Reversed by Neutralizing BDNF

**Figure 6:**
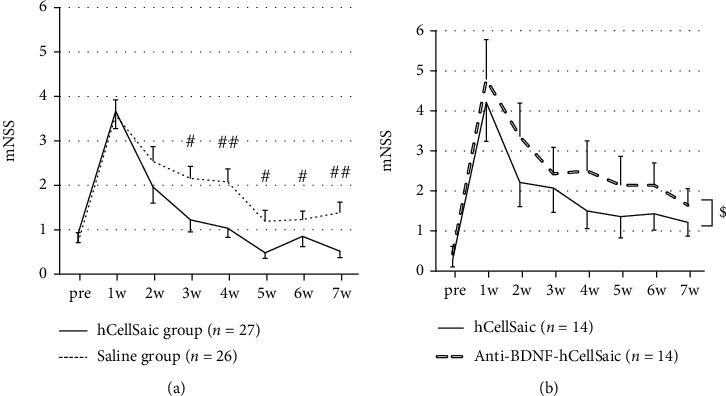
Human-derived CellSaic for the ICH model in immunodeficient rats and the reversal of efficacy by BDNF neutralization through CellSaic. hCellSaic were transplanted into the hollow cavity of immunodeficient rats. The hCellSaic group showed significant neurological recovery from 2 weeks after transplantation compared with the saline transplanted group (a). hCellSaic group was compared with the anti-BDNF-hCellSaic (BDNF inhibitor added to hCellSaic) group. The anti-BDNF-hCellSaic group with BDNF released from CellSaic was blocked, showing significantly less recovery compared to the normal hCellSaic group after transplantation via two-way analysis of variance. Data are presented as the mean ± SE. #*p* < 0.05 vs. saline group; ##*p* < 0.01 vs. saline group; $*p* < 0.05 (two-way analysis of variance).

hCellSaic made from human bone marrow-derived MSC (hBMSC) was also evaluated for future clinical application. Complete hematoma absorption and cavity formation was defined as chronic ICH, and rats were serially euthanized for up to 1 week, and hematomas were absorbed, and hematoma cavities were formed until 7 d after ICH by using ouabain (Supplementary Figure IB). hCellSaics or saline were transplanted 7 d after hemorrhage, and functional recovery was serially monitored, wherein the hCellSaic group showed significantly better recovery at 2, 3, 4, 5, and 6 weeks after the transplantation than the saline group (*p* = 0.0103, 0.0054, 0.0275, 0.0421, and 0.0035, respectively; Figure 6(a)). To validate the mode of action of stem cell treatment, neurotrophic factor inhibitors were administered to diminish their function. The releasing ability of BDNF, GDNF, and HGF was inhibited by antibody, where BDNF and GDNF were successfully inhibited (Supplementary Figure II), and BDNF was then selected for further *in vivo* study. After transplantation, the anti-BDNF-hCellSaic group showed significantly less recovery than the hCellSaic group (*p* = 0.0303; Figure 6(b)), indicating that BDNF was, at least to some extent, responsible for neural recovery.

## 4. Discussion

In the present study, we found that CellSaic transplantation into the hollow cavity contributed to neurological improvement in two different animal models of chronic ICH. We demonstrated that CellSaic secreted higher amounts of trophic factors and achieved better cell survival than BMSC alone in *in vitro* and *in vivo* conditions. Furthermore, histochemistry and nuclear imaging revealed that CellSaic administration alleviated neuronal degeneration and that BDNF was partially responsible for recovery.

Currently, most experimental and clinical cell therapies for stroke treatment adopt isolated stem cells for transplantation through the intravenous or intracerebral routes [23]. Intravenous transplantation is often preferred in acute settings since the transplanted cells can ameliorate local and systemic inflammation [5]. In contrast, intracerebral transplantation is considered advantageous after the subacute phase of stroke. In these phases, inflammation is mostly settled, and reorganization of the neuronal network is required for the function of stem cells. However, cell survival and engraftment are among the most important obstacles to overcome for recovery in intracerebral transplantation, because most stem cells require cell-cell or cell-extracellular matrix contact to avoid cell death, often referred to as anoikis. Cell clusters developed only from isolated cells face the disadvantage of central necrosis, which results from poor nutrition in the core of the cluster with failure of metabolic waste removal [24]. In that circumstance, numerous biomaterials possessing cell attachment capacity have been designed as bioscaffolds [25].

RCP, developed by our group, is a recombinant peptide consisting of alpha-1 sequence of 12 RGD motifs in a single molecule and enables high cell attachment properties. It is produced by the yeast *Pichia pastoris* and possesses no risk of animal-related infection, thus possessing zero risk relative to that of animal-obtained collagen [10]. Stem cells compounded with RCP (CellSaic) have been shown to be effective in subcutaneous transplantation in a diabetes mellitus model and intraperitoneal injection in a colitis model [10, 14], and better cell survival and engraftment with angiogenesis are considered to be the reason for the superiority of this product. In this study, we also found that BMSC compounded with RCP exhibited higher neurotrophic factor release capacity and cell survival/engraftment, which will be important for clinical application when a shortage of oxygen and nutrition are expected at the injection site.

In this study, intracerebral transplantation of CellSaic into a hollow cavity showed favorable results in different animal models and types of cell origin. These results strengthen the therapeutic advantage of CellSaic since intracavity transplantation can standardize the transplantation procedures in clinical trials, which are essential for evaluating the efficacy of the drug by eliminating the technical aspect of the surgeon. We have also proved successful transplantation without immunosuppressants, which may be beneficial for eliminating immunosuppressant drugs that may cause compromised situations for transplant patients. It has been suggested that major histocompatibility complex matching improves the engraftment rate [26], and autologous transplantation may further improve the recovery. In addition to better cell viability, CellSaic showed better secretion potential of neurotrophic factors, including BDNF. We have demonstrated that CellSaic transplantation increases the local BDNF level, which might be caused by enhanced cell viability. Further, we show that the effect of CellSaic is neutralized by blocking BDNF secretion. These results strongly support the notion that BDNF is a key factor in CellSaic therapy for ICH.

In previous studies, other trophic factors have also been reported to correlate with neurological recovery after ICH. Liu et al. demonstrated that HGF-transfected MSC transplantation for ICH achieved better functional improvement than nontransfected MSC, and they concluded that HGF had therapeutic potential through nerve fiber remyelination and axonal regeneration [27]. Likewise, Yang et al. reported that GDNF-transfected BMSC transplantation for ICH caused significantly lowered apoptosis and consequent better functional recovery [28]. Similarly, Deng et al. have suggested that GDNF-transfected MSC have the potential to promote neurological recovery [29]. Thus, HGF and GDNF are also important trophic factors in stem cell therapy for ICH. The difference can be attributed to the methodology, including timing of cell administration, animal model, and functional tests; further study is warranted to elucidate the relationship between recovery and trophic factors.

In this study, we also discovered that CellSaic could secrete more exosomes than solo-BMSC in an OGD environment. In recent years, exosomes have been recognized to be one of the positive factors of stem cell therapies [30]. Although the mechanism(s) of exosomes remains unclear, some previous reports have suggested that exosomes or their contents, including microRNAs (miRNA), influence the improvements made by stem cell therapies after hemorrhagic stroke. Otero-Ortega et al. showed that ICH rats receiving exosomes derived from MSC demonstrated better functional recovery than control rats [31]. Shen et al. suggested that exosomes derived from miRNA-133b-transfected MSC prevented apoptosis and necrosis in brain tissue [32]. Wang et al. described that miRNA-126-modified MSC had the ability to protect blood-brain barrier integrity and achieve a better functional outcome [33]. We did not examine in detail which substance(s) in the exosomes plays an important role; however, we believed that enhanced secretion of exosomes from CellSaic showed favorable effects against chronic ICH.

Although not statistically significant, we found that CellSaic transplantation showed a higher trend of uptake of iomazenil ligand in the affected hemisphere compared with the solo-BMSC transplantation. Iomazenil ligand used for ^23^I-IMZ SPECT selectively binds to central benzodiazepine receptors and is reported to reflect neuronal cell density and viability [34]. Therefore, this result indicates cessation of neurodegradation in the chronic phase and/or facilitation of neuroregeneration. We found that CellSaic transplantation showed higher uptake in the ICH marginal area. This is in line with our previous ischemic stroke study [20], in which intracerebral injection of stem cells ameliorated the neurons located in the border zone where blood supply or other aspects interfere with neuronal survival. Together with the data on brain atrophy, CellSaic was able to cease the negative cascade of cell damage, such as Wallerian degeneration, which can take place even in the chronic phase [35]. Neuroregeneration can be another explanation for the increase in iomazenil uptake. Stem cell therapy is reported to upregulate the neuroregeneration of immature neuronal cells located around the subventricular zone, and Vaquero et al. reported that intracerebral injection of stem cells with their scaffold facilitated neuroregeneration, which was confirmed by immunostaining [7, 8].

Immunodeficient animals that received CellSaic composed from human-derived MSC also showed better neurological recovery compared to those that received human-derived MSC alone. It is quite important when conducting clinical research to show the quality of human-derived products. Human-derived cell products may exhibit different characteristic from the analogous animal-derived product; moreover, it is necessary to confirm the absence of tumor formation from the transplanted cells [36]. One clue to the quality control of human-derived MSC is the use of induced pluripotent stem cells (hiPSCs). MSC derived from hiPSCs can be expanded with uniform quality, which makes it easier to control cell quality and quantity. Previous studies suggest the usefulness of this cell type as it presents immunomodulatory properties and releases various trophic factors [37, 38] and can be an option for clinical use [39].

There are several limitations to our study. First, our ICH models may not be regarded as complete chronic models considering the time course after inducing ICH. We defined empty cavity formation as a chronic state; however, it is unknown whether this state fully resembles the one in human chronic ICH. CellSaic transplantation at a later stage may be required to strengthen the efficacy of CellSaic. Second, transplantation at different locations was not examined in this study. Transplanting CellSaic into the normal brain near the hollow cavity may be one option; however, we did not select this route because the size of CellSaic (approximately 200–250 *μ*m) is much larger than that of solo-BMSC (approximately 15–25 *μ*m), and transplantation of CellSaic in the normal brain may result in mass effect that is detrimental to neural recovery. Third, we found that TrkB/Fc chimera protein decreased the expression of BDNF in BMSC and reversed the functional recovery of CellSaic. However, human TrkB/Fc chimera protein may also block the binding of other important neurotrophic factors, including neurotrophin-4/5. Since we did not evaluate the expression of neurotrophin-4/5, it is believed that the expression of neurotrophin-4/5 is also decreased in the CellSaic, and it may also account for the mechanism of CellSaic.

## 5. Conclusions

CellSaic, a BMSC and recombinant peptide compound, demonstrated a high capacity for releasing neurotrophic factors, and administering CellSaic into the hollow cavity of chronic ICH facilitated neurological functions, presumably by releasing BDNF. CellSaic preserved the neurons around the cavity and prevented brain atrophy. This discovery could be crucial to improvements in the treatment and outcomes of chronic ICH and requires further study.

## Data Availability

The data used to support the findings of this study are included within the article.

## Supplementary Materials

Supplementary 1 Supplementary material and method: full description of the methods including the experimental design.

Supplementary 2 Supplementary Figure 1. Hematoma cavity formation in the collagenase and ouabain models. Intracerebral hemorrhages (ICHs) were absorbed and became hollow cavities after 2 weeks in the collagenase model (A), and the hematoma cavities were completely formed until 7 d after ICH in the ouabain model (B).

Supplementary 3 Supplementary Figure 2. Blocking of neurotrophic factors. Brain-derived neurotrophic factor (BDNF) and glial cell line-derived neurotrophic factor (GDNF) secreted from CellSaics were suppressed by their inhibitors. The concentrations of BDNF (A) and GDNF (B) were inhibited by recombinant human TrkB Fc chimera protein (BDNF inhibitor) and human GDNF antibody, and the suppression continued for at least 5 d after adding the inhibitors, although the antibodies were removed. In contrast, hepatocyte growth factor (HGF) (C) was only partially inhibited by the human HGF antibody.

Supplementary 4 Supplementary video: cell survival and migration after OGD exposure for CellSaic.
