# Supplementary material for: Intracerebral Transplantation of Mesenchymal Stromal Cell Compounded with Recombinant Peptide Scaffold against Chronic Intracerebral Hemorrhage Model
Source: Stem Cells Int. 2022 Jul 31;2022:8521922. doi: 10.1155/2022/8521922 (PMC9372516; doi:10.1155/2022/8521922)
Supplement: Supplementary 1 — Supplementary material and method: full description of the methods including the experimental design. [file 8521922.f1.docx]

**Isolation and culture of rat BMSC (rBMSC)**

Rats were euthanized and bone marrow was aseptically collected from femurs using Dulbecco’s Modified Eagle Medium (DMEM) - High Glucose (Nacalai Tesque Inc., Kyoto, Japan) supplemented with 2% penicillin-streptomycin mixed solution (P/S; Nacalai Tesque Inc., Kyoto, Japan), 10% fetal bovine serum (FBS; Life Technologies Co., Carlsbad, CA, USA), and 10% heparin sodium. The collected marrow cells were plated in a cell culture flask coated with collagen I (Corning Inc., Glendale, AZ, USA), and cultured in an incubator maintained at 37°C in a humidified atmosphere containing 5% CO_2_, with DMEM (high glucose) containing 10% FBS and 2% P/S. After 24 h, the non-adherent cells were removed, and the adherent cells continued to be cultured and passaged. The cells were passaged more than three times and used for subsequent experiments.

**In vitro assessment of rCellSaic**

rCellSaic and rBMSC were incubated at 37°C in an atmosphere containing 1% O_2_ and 5% CO_2_ using an InvivO2 300 Hypoxia Workstation (Baker Ruskinn, Bridgend, UK). Before incubation, DMEM without glucose (Nacalai Tesque Inc., Kyoto, Japan) was placed in the hypoxia cabinet for a day to remove dissolved oxygen. The medium of rCellSaic and rBMSC was replaced with the prepared medium just before incubation. Four hundred rCellSaic (1×10^6^ BMSC equivalent; n=6) or 1×10^6^ BMSCs (n=6) with 10 mL medium were dispensed into 15 mL tubes, and then incubated in an OGD environment for 52 h. After incubation, the culture supernatants and precipitated rCellSaic/rBMSC were collected separately.

Trophic factor secretion from rCellSaic and rBMSC was evaluated by enzyme-linked immunosorbent assay (ELISA). The collected supernatants were used to measure the following trophic factors: brain-derived neurotrophic factor (BDNF), hepatocyte growth factor (HGF), vascular endothelial growth factor (VEGF), and glial cell line-derived neurotrophic factor (GDNF). They were quantified using commercially available ELISA kits (total BDNF, rat HGF, and rat VEGF; R&D Systems, Inc., MN, USA; rat GDNF; RayBiotech, Inc., Peachtree Corners, GA, USA) according to the manufacturer’s protocols.

Exosomes were prepared from the collected culture supernatants of CellSaic and BMSC; 1.5 mL of the supernatant was sequentially centrifuged at 2,000×g for 10 min and 10,000×g for 30 min to remove dead cells and debris. Subsequently, they were spun again at 100,000×g for 70 min at 4°C to obtain exosomes as pellets. The pellets were resuspended in 100 µL phosphate buffered saline, and the exosomal particle size and density were analyzed using a qNano System (Izon Science, Ltd., Christchurch, New Zealand).

After exposure to OGD conditions, the CellSaic/BMSC were re-cultured at 37°C in an atmosphere containing 21% O_2_ and 5% CO_2_ to examine the cell growth capacity. The medium was replaced with DMEM containing high glucose. CellSaic/BMSC were fed fresh medium every 3 d. For CellSaic, time-lapse imaging was performed using an All-in-One Fluorescence Microscope BZ-X700 (Keyence Co., Osaka, Japan) to chronologically observe cell proliferation. After 7 d, the number of living cells was manually counted in the bright field under a magnification of 40×.16 Five non-overlapped fields were randomly selected, and their average number was calculated.

**Chronic ICH model for rCellSaic**

Eighty-six 8-week-old male Sprague-Dawley (SD) rats (CLEA Japan, Inc., Tokyo, Japan) were used for the rCellSaic experiments. Animals were housed at 23±3°C temperature, 50±20% humidity, and a 12-h light-dark cycle with free access to food and water. They were acclimatized to the environment for a week before the experiments.

ICH models: To create the collagenase model, SD rats were stereotactically infused with bacterial collagenase into the right internal capsule, as previously reported with minor modifications ^1^. After the induction of general anesthesia with 5% isoflurane in 70% N_2_O and 30% O_2_ gas, followed by maintenance anesthesia with 1.5–2% isoflurane in 70% N_2_O and 30% O_2_ gas, the rats were placed in a small animal stereotactic instrument (Model 900; David Kopf Instruments, Tujunga, CA, USA) in the prone position, and their skulls were exposed via a midline skin incision. Through a burr hole created by a dental drill, a 26-gauge Hamilton syringe needle was inserted into the right internal capsule (coordinates: 2.0 mm posterior, 3.7 mm lateral to the bregma, and 6.0 mm ventral from the dura mater). Subsequently, 2.5 µL saline containing 0.2 U type IV collagenase (Sigma-Aldrich, St. Louis, MO, USA) was injected for 5 min using a micro-infusion pump (Model KDS-310; Muromachi Kikai Co., Ltd., Tokyo, Japan). The needle was left in place for another 5 min, and then slowly removed. Finally, the skin wound was sutured.

We defined chronic ICH as a condition in which the induced hematoma was completely absorbed and the cavity became empty. In order to validate when ICH becomes a chronic ICH, rats were euthanized to collect their brains 3, 7, and 14 d after ICH, and the absorption rate of hemorrhage was serially analyzed.

**Transplantation of rCellSaic**

rCellSaic, rBMSC, or saline was transplanted into the hollow cavity two weeks after ICH. Sixty-two Sprague-Dawley rats were randomly assigned to three groups: rCellSaic group (n=24), rBMSC group (n=18), and saline group (n=20). The animals did not receive immunosuppressant drugs. They were anesthetized as described above and underwent stereotactic injection of rCellSaic equivalent to 1.0×10^6^ of rBMSC (10 μL) using a 21-gauge needle, 10 μL of saline containing 1.0×10^6^ rBMSC, or 10 μL of saline using a 21-gauge Hamilton syringe into the cavity (coordinates: 2.0 mm posterior, 3.7 mm lateral to the bregma, and 5.0 mm ventral from the dura matter). They were injected for a few minutes, and the needle was left in place for another 5 min.

**Functional and histological assessment**

Neurological function was assessed using the modified neurological severity score (mNSS) before and at 1, 7, 14, 21, 28, 35, and 42 d after ICH as previously reported with minor modifications ^2^. The mNSS is a comprehensive neurological scale with scores ranging from 0 (normal) to 18 (most severe deficit), and consists of a motor test (0–6 points), sensory test (0–2 points), beam balance test (0–6 points), and reflex absence/abnormal movement (0–4 points). Because our ICH models usually circled toward the non-paretic side, 2 points in “Placing rat on the floor” was changed from “Circling toward the paretic side” to “Circling toward the non-paretic side”. For the beam balance test, a square bar with a width of 2 cm was used.

For histopathological assessment, brains were collected at 42 d after ICH (rCellSaic group, n=13; rBMSC group, n=8; saline group, n=13) as previously described ^3^. The rats were anesthetized using 5% isoflurane in 70% N_2_O and 30% O_2_ gas, and transcardially perfused with cold saline followed by 4% paraformaldehyde (PFA). After decapitation, the brains were gently collected and fixed with 4% PFA for 24 h. Thereafter, they were sliced into 2-mm thick coronal sections and embedded in paraffin. They were finally cut using a manual microtome (Leica RM2125 RTS, Leica Biosystems, Nussloch, Germany) to 5-µm. Brain Hematoxylin and eosin (H&E) staining was performed to assess brain atrophy. The brain coronal sections were located 4 mm posterior to the bregma, where the maximum diameters of most ICH were soaked in hematoxylin solution (Muto Pure Chemicals Co., Ltd., Tokyo, Japan) for 5 min and eosin solution (Muto Pure Chemicals Co., Ltd., Tokyo, Japan) for 4 min. For each slide, the ipsilateral and contralateral parenchymal areas were measured using ImageJ software (ImageJ 1.52a, National Institutes of Health, Bethesda, MD, USA). The parenchymal area and brain atrophy rate were calculated using the following formula:

Parenchymal area (mm^2^) = hemispheric area - (hematoma area + ventricular area)

Brain atrophy rate (%) = [(contralateral parenchymal area – ipsilateral parenchymal area) / contralateral parenchymal area] × 100

Immunohistochemistry was performed for GFP to identify whether the transplanted CellSaics or BMSCs exist in the brain. Coronal brain sections were incubated with anti-GFP antibody (1:1,000, ab6556; Abcam, Cambridge, UK) for 1 h at room temperature. The sections were then treated with Histofine Simple Stain MAX-PO (Nichirei Biosciences Inc., Tokyo, Japan) for 30 min, and subsequently reacted with the ImmPACT VIP substrate (SK-4605; Vector Laboratories, Burlingame, CA, USA) for 5 min.

To substantiate whether the trophic factors secreted from CellSaic could be detected from the CellSaic-transplanted brain, BDNF levels in brain tissue were measured. Brains were collected from Sprague-Dawley rats 3 d after transplantation (rCellSaic group, n=7; rBMSC group, n=6; saline group, n=7). After induction of general anesthesia, the rats were transcardially perfused with cold saline and decapitated. The brains were gently collected, sliced into 2-mm thick coronal sections on ice, and immediately stored at −80°C. The ipsilateral and contralateral brain hemispheric sections located 2–4 mm posterior to the bregma were used for samples, and separately homogenized in RIPA lysis buffer with protease inhibitor (Santa Cruz Biotechnology, Inc., Dallas, TX, USA) using a bead mill benchtop homogenizer (Shakeman 3; BioMedical Science Co., Ltd, Tokyo, Japan). The homogenates were centrifuged twice at 12,000×g for 10 min, and the supernatants were collected for analysis. The concentration of total protein in each sample was measured using a protein assay kit (Pierce BCA Protein Assay Kit; Thermo Fisher Scientific, Waltham, MA, USA), and that of BDNF was quantified using total BDNF ELISA kits (R&D Systems, Inc., Minneapolis, MN, USA) according to the manufacturer’s protocols. The BDNF concentration was then divided by the total protein concentration to correct it to the value per unit protein weight. The BDNF increment due to transplantation was finally evaluated by taking the ratio of the ipsilateral and contralateral corrected BDNF concentrations as follows:

Corrected BDNF = BDNF concentration / total protein concentration

BDNF increasing rate = ipsilateral corrected BDNF / contralateral corrected BDNF

Eight Sprague-Dawley rats (rCellSaic group, n=4; rBMSC group, n=4) were used to evaluate the integrity of neural receptors using I-123-iomazenil (123I-IMZ) single photon emission computed tomography (SPECT) as previously reported ^4^. 123I-IMZ (Benzodine; Nihon Medi-Physics Co., Ltd., Tokyo, Japan) uptake around the hematoma cavity was evaluated using a small animal imaging system (Inveon SPECT/CT; Siemens Medical Solutions USA, Inc., Knoxville, TN, USA). SPECT/CT imaging studies were performed 1 and 4 weeks after transplantation. Rats were intravenously injected with approximately 120 MBq of 123I-IMZ via the tail vein. One hour after the injection, the rats were anesthetized using 2% isoflurane in air and scanned by SPECT for 90 min, followed by CT for 15 min. The conditions used for the SPECT scans were as follows: radius of rotation, 35 mm; number of projections, 60; scan time, 180 seconds/view; collimator, single-pinhole of 2.0 mm aperture. The acquired data were reconstructed using the 3-dimensional ordered subset expectation maximization method with 16 iterations per 6 subsets. The reconstructed images were analyzed using the Inveon Research Workplace software (Siemens Medical Solutions USA, Inc.). Regions of interest (ROIs) of 1 mm thickness were placed on the ipsilateral and contralateral hemispheres in the coronal sections 4 mm posterior to the bregma. ROIs on the contralateral hemisphere were defined and then horizontally inverted to define ROIs in the ipsilateral hemisphere. The ROIs defined in the first scan were transferred to the ROIs in the second scan. The radioactivity in each ROI was quantified using the Inveon Research Workplace software, and the ipsilateral to contralateral ratios were calculated. The variation rates of the ipsilateral/contralateral ratio from the first scan (1 week after the transplantation) to the second scan (4 week after transplantation) were calculated.

**Preparation of human BMSC**

The frozen cryovials of human MSC (Lonza, Basel, Switzerland) were thawed and cells were re-cultured in a cell culture flask (Corning Inc.) at 37°C and 5% CO_2_ in Mesenchymal Stem Cell Growth Medium BulletKit (Lonza, Basel, Switzerland). After 24 h, the non-adherent cells were removed, and the adherent cells continued to be cultured and passaged. After three passages, the cells were used for the subsequent experiments.

**Chronic ICH model for hCellSaic transplantation**

F344/NJcl-rnu/rnu rats underwent stereotactic infusion of ouabain into the right internal capsule, as previously reported with minor modifications ^1, 5^. General anesthesia was induced and maintained as previously described. The rats were placed on a small animal stereotactic instrument (Model SR-5R-HT; David Kopf Instruments) in the prone position, and their skulls were exposed via a midline skin incision. Through a burr hole created by a dental drill, a 27-gauge needle connected to a Hamilton syringe was inserted into the right internal capsule (coordinates: 1.5 mm posterior, 3.5 mm lateral to the bregma, and 6.0 mm ventral from the dura mater). Subsequently, 3.0 µL of 7.5 mM ouabain octahydrate solution (Sigma-Aldrich) was injected for 5 min using a micro-infusion pump (Model Nexus3000; ISIS Co., Ltd., Tokyo, Japan). The needle was left in place for another 5 min, and then slowly removed. Finally, the skin wound was sutured. A total of 81 rats were included in further experiments.

One week after ICH, 53 F344/NJcl-rnu/rnu rats were randomly assigned to two groups: hCellSaic group (n=27) and saline group (n=26). They were injected with hCellSaics or saline into the hematoma cavity to clarify the effect of hCellSaic as a xenograft. The animals in the immunodeficiency model did not receive immunosuppressant drugs. They were anesthetized as described above and stereotactically injected with hCellSaics, which was equivalent to 4.8×10^5^ of hBMSC (10 μL) using a 21-gauge needle or 10 μL of saline using a 21-gauge Hamilton syringe into the cavity (coordinates: 1.5 mm posterior, 3.5 mm lateral to the bregma, and 5.0 mm ventral from the dura matter). CellSaics or saline were injected for a few minutes, and the needle was left in place for another 5 min to avoid leakage.

**Neutralizing the trophic factor secretion potential of MSC**

Neurotrophic factor secretion was suppressed in CellSaics to evaluate its contribution to functional recovery. For BDNF, HGF, and GDNF inhibition, recombinant human TrkB Fc chimera protein (1 µg/mL) (688-TK-100; R&D Systems, Inc., MN, USA), human HGF antibody (3 µg/mL) (MAB294-500; R&D Systems, Inc., MN, USA), and human GDNF antibody (1 µg/mL) (MM0308-12F9; Novus Biologicals, Inc., CO, USA) were co-cultured with CellSaics for 24 h respectively17. IgG isotype control (1 µg/mL) (MAB002; R&D Systems, Inc.) was used as a negative control. The neutralizing antibodies were then removed, and CellSaics were fed with fresh medium. Medium exchange was performed 1 and 4 d after antibody removal. Before the medium exchange, the culture supernatants were collected to evaluate whether specific trophic factors were accurately inhibited. Neurotrophic factors were quantified using commercially available ELISA kits as previously mentioned.

Transplantation of hCellSaic and anti-BDNF-hCellSaic

One week after ICH, 28 F344/NJcl-rnu/rnu rats were randomly assigned to two groups; the hCellSaic group (n=14) and anti-BDNF-hCellSaic group (n=14). They were injected with hCellSaics or anti-BDNF-hCellSaic into the hematoma cavity to clarify whether BDNF was related to the effect of CellSaic. The animals did not receive immunosuppressant drugs. They were anesthetized as described above and stereotactically underwent injection of hCellSaics or anti-BDNF-hCellSaic, which were equivalent to 4.8×10^5^ of hBMSC (10 μL) using a 21-gauge needle into the cavity (coordinates: 1.5 mm posterior, 3.5 mm lateral to the bregma, and 5.0 mm ventral from the dura matter). They were injected for a few minutes, and the needle was left in place for another 5 min.

References

1. Liu Y, Lu G, Su XW, Ding T, Wang WL, Li YM, et al. Characterization of axon damage, neurological deficits, and histopathology in two experimental models of intracerebral hemorrhage. *Front Neurosci*. 2018;12:928

2. Chen J, Li Y, Wang L, Zhang Z, Lu D, Lu M, et al. Therapeutic benefit of intravenous administration of bone marrow stromal cells after cerebral ischemia in rats. *Stroke*. 2001;32:1005-1011

3. Wang Z, Higashikawa K, Yasui H, Kuge Y, Ohno Y, Kihara A, et al. Fty720 protects against ischemia-reperfusion injury by preventing the redistribution of tight junction proteins and decreases inflammation in the subacute phase in an experimental stroke model. *Transl Stroke Res*. 2020

4. Saito H, Magota K, Zhao S, Kubo N, Kuge Y, Shichinohe H, et al. 123i-iomazenil single photon emission computed tomography visualizes recovery of neuronal integrity by bone marrow stromal cell therapy in rat infarct brain. *Stroke*. 2013;44:2869-2874

5. Shichinohe H, Yamauchi T, Saito H, Houkin K, Kuroda S. Bone marrow stromal cell transplantation enhances recovery of motor function after lacunar stroke in rats. *Acta Neurobiol Exp (Wars)*. 2013;73:354-363
