# Supplementary figures and images for: Intracerebral Transplantation of Mesenchymal Stromal Cell Compounded with Recombinant Peptide Scaffold against Chronic Intracerebral Hemorrhage Model
Source: Stem Cells Int. 2022 Jul 31;2022:8521922. doi: 10.1155/2022/8521922 (PMC9372516; doi:10.1155/2022/8521922)

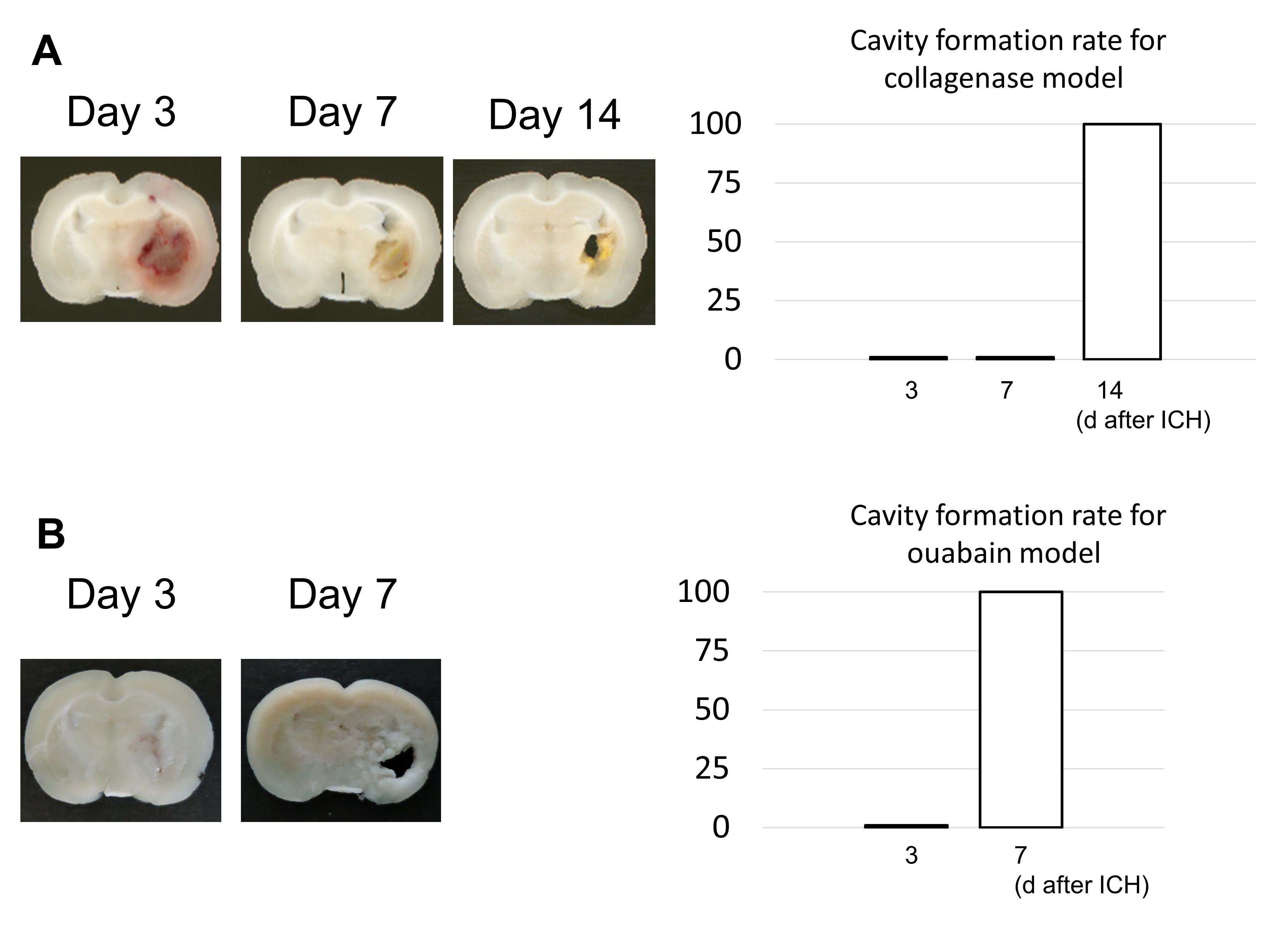

Supplement: Supplementary 2 — Supplementary Figure 1. Hematoma cavity formation in the collagenase and ouabain models. Intracerebral hemorrhages (ICHs) were absorbed and became hollow cavities after 2 weeks in the collagenase model (A), and the hematoma cavities were completely formed until 7 d after ICH in the ouabain model (B). [file 8521922.f2.JPG]

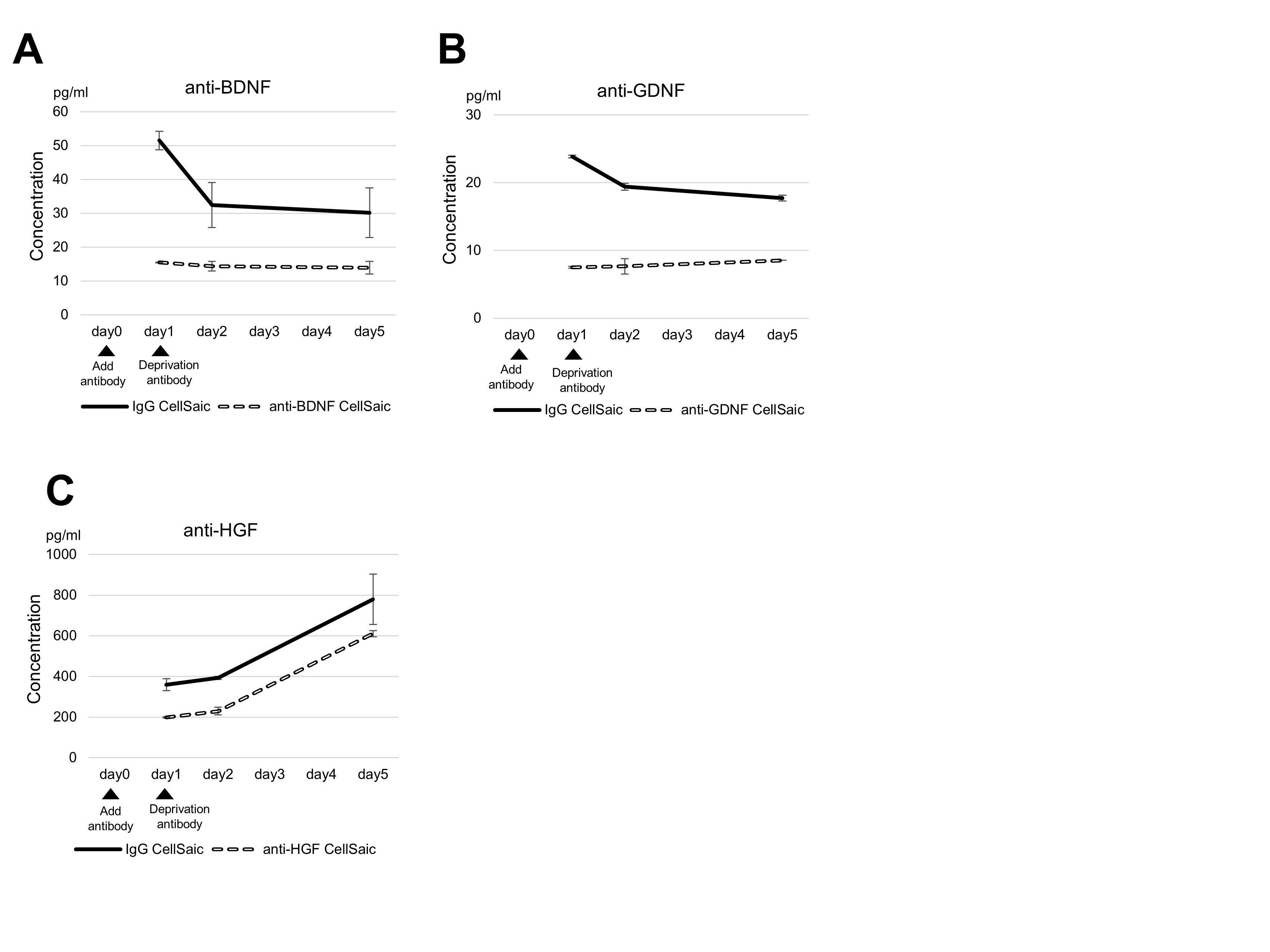

Supplement: Supplementary 3 — Supplementary Figure 2. Blocking of neurotrophic factors. Brain-derived neurotrophic factor (BDNF) and glial cell line-derived neurotrophic factor (GDNF) secreted from CellSaics were suppressed by their inhibitors. The concentrations of BDNF (A) and GDNF (B) were inhibited by recombinant human TrkB Fc chimera protein (BDNF inhibitor) and human GDNF antibody, and the suppression continued for at least 5 d after adding the inhibitors, although the antibodies were removed. In contrast, hepatocyte growth factor (HGF) (C) was only partially inhibited by the human HGF antibody. [file 8521922.f3.JPG]
